# Researches on the Influence of the Nerves in Inflammation

**Published:** 1858-05

**Authors:** H. Snellen


### RESEARCHES ON THE INFLUENCE OF THE NERVES IN INFLAMMATION.—BY H. SNELLEN.

This interesting work is recapitulated in the following conclusions:

"1. Excitation of the nerves of sensibility determines, by reflex action, the contraction of the bloodvessels of the neighboring parts."

It may determine contractions of very distant bloodvessels: for example, the irritation of one hand by cold will cause the contraction, by reflex action, of the bloodvessels of the other hand, as M. Tholozan and myself had already found some six or seven years ago.

"2. The contraction of the bloodvessels is followed after a little time by their dilatations."

"There is not any proof that in the walls of the vessels there exists any other nerves than those that cause their contraction."

"4. The nerves of the bloodvessels modify nutrition, by acting upon the calibre of these conduits."

"5. The inflammatory process does not consist essentially in a modification of the nervous influence."

"6. The inflammation of the cornea that manifests itself after section of the trifacial nerve, is not the direct result of absence of nervous influence in the ganglion of Gasserius."

This last point, which is the most important, is established by Snellen, by the absence of alteration in the eye, or, at least, in the cornea after section of the trifacial nerve, when he took care to cover the eye, holding the lids shut and covered by the ear of the animal. Under these circumstances, the drying of the cornea could not occur, and the light could not irritate the eye. I exhibited to M. Magendie, about nine years ago, that in frogs, after ablation of the medulla oblongata and Gasserius' ganglion, the two eyes remained in a perfectly normal condition entire months, so long as the animals were kept in a very cold, dark, and humid atmosphere. E. B.-S.

